# Gene expression profiling following constitutive activation of MEK1 and transformation of rat intestinal epithelial cells

**DOI:** 10.1186/1476-4598-5-63

**Published:** 2006-11-17

**Authors:** Koga Komatsu, F Gregory Buchanan, Michiro Otaka, Mario Jin, Masaru Odashima, Yohei Horikawa, Sumio Watanabe, Raymond N DuBois

**Affiliations:** 1 Department of Gastroenterology, Honjo Daiichi General Hospital, Yurihonjo, Japan; 2 Department of Gastroenterology/Internal Medicine, Akita University School of Medicine, Akita, Japan; 3 Departments of Medicine and Cancer Biology, Vanderbilt-Ingram Cancer Center, Nashville, USA

## Abstract

**Background:**

Constitutive activation of MEK1 (caMEK) can induce the oncogenic transformation of normal intestinal epithelial cells. To define the genetic changes that occur during this process, we used oligonucleotide microarrays to determine which genes are regulated following the constitutive activation of MEK in normal intestinal epithelial cells.

**Results:**

Microarray analysis was performed using Affymetrix GeneChip and total RNA from doxycycline inducible RIEtiCAMEK cells in the presence or absence of doxycycline. MEK-activation induced at least a three-fold difference in 115 gene transcripts (75 transcripts were up-regulated, and 40 transcripts were down-regulated). To verify whether these mRNAs are indeed regulated by the constitutive activation of MEK, RT-PCR analysis was performed using the samples from caMEK expressing RIE cells (RIEcCAMEK cells) as well as RIEtiCAMEK cells. The altered expression level of 69 gene transcripts was confirmed. Sixty-one of the differentially expressed genes have previously been implicated in cellular transformation or tumorogenesis. For the remaining 8 genes (or their human homolog), RT-PCR analysis was performed on RNA from human colon cancer cell lines and matched normal and tumor colon cancer tissues from human patients, revealing three novel targets (rat brain serine protease2, AMP deaminase 3, and cartilage link protein 1).

**Conclusion:**

Following MEK-activation, many tumor-associated genes were found to have significantly altered expression levels. However, we identified three genes that were differentially expressed in caMEK cells and human colorectal cancers, which have not been previously linked to cellular transformation or tumorogenesis.

## Background

Mitogen-activated protein kinases (MAPKs) are serine-threonine kinases activated by phosphorylation of specific amino acids in response to extracellular stimuli and have been shown to play an important role in tumorigenesis [1-8]. The first member of this family to be characterized was the extracellular signal-regulated protein kinase (ERK), which is phosphorylated and activated by MAPK/ERK kinase (MEK) [1,2]. The MEK-ERK signaling pathway is one of the downstream targets of oncogenic mutations in *ras *[1,2] and the increased activity of MEK has been identified in many human malignancies, including colorectal cancer [9]. Constitutive activation of MEK1 signaling can induce the oncogenic transformation of fibroblast [10-12], kidney [13], mammary [14], and intestinal epithelial cells [8,15]. We recently reported that the oncogenic potential of MEK in intestinal epithelial cells was mediated by cyclooxygenase-2 (COX-2) [8]. COX-2 and its derived prostaglandins are also thought to be involved in the development and progression of colorectal cancer [16,17]. The MEK-ERK cascade has been reported to induce increased tumor invasiveness [18,19], pro-cell cycle properties [8,20], angiogenesis [21], anti-apoptosis [8,22], and resistance to some anti-cancer agents [23,24]. However, the precise role of MEK-ERK signaling in intestinal carcinogenesis remains unknown.

In the past few years, newly developed technologies such as gene microarrays [25] have enabled the determination of molecular differences between normal and transformed cells at a genome-wide level. However, since most of these analyses were performed using bulk tissue samples that are composed of multiple cell lineages, the specific roles of identified genes during tumorigenesis are still under investigation. Therefore, the information obtained from a single cell before and after activation of a key signaling pathway during transformation may be a useful strategy for identifying novel targets. We previously established tetracycline regulated constitutively activated MEK1 (caMEK) expressing normal rat intestinal epithelial cells (RIEtiCAMEK cells), and reported that caMEK could induce the transformation of RIE and IEC-6 cells [8]. To clarify the oncogenic potential of MEK-ERK signaling and to identify novel targets of colonic carcinogenesis, we sought to determine the genes involved in caMEK-mediated transformation by gene microarray and RT-PCR analysis.

## Results

### Microarray results from RIEtiCAMEK cells

Total RNA from RIEtiCAMEK cells with/without doxycycline (DOX) following treatment with 5 mM sodium butyrate (NaB) for 48 hours were submitted for microarray analysis. RIEtiCAMEK cells express high levels of caMEK upon removal of DOX from the culture media and in the presence of NaB. One hundred-fifteen genes were observed (75 genes showed increased expression, while 40 genes were down regulated) with at least a three-fold difference in expression (data not shown).

### Confirmation of microarray results by RT-PCR analysis

**Figure 1:**
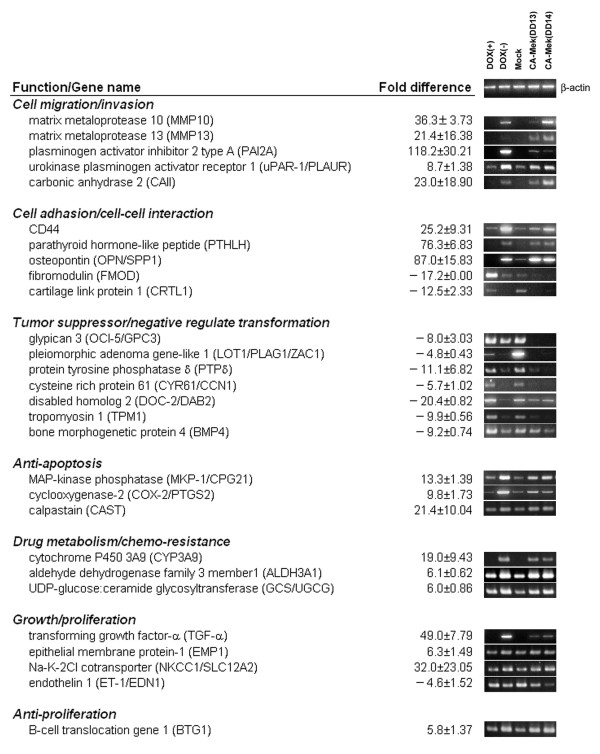
Altered expression levels of caMEK-regulated genes involved in cell migration/invasion, cell adhesion, tumor suppression, anti-apoptosis, drug metabolism, and growth/proliferation. The microarray results from caMEK expressing cells (DOX(-)) compared to normal cells (DOX(+)) are expressed as fold difference ± S.D. Differentially expressed genes were verified through RT-PCR analysis. β-actin was used to indicate equal template in each lane.

**Figure 2:**
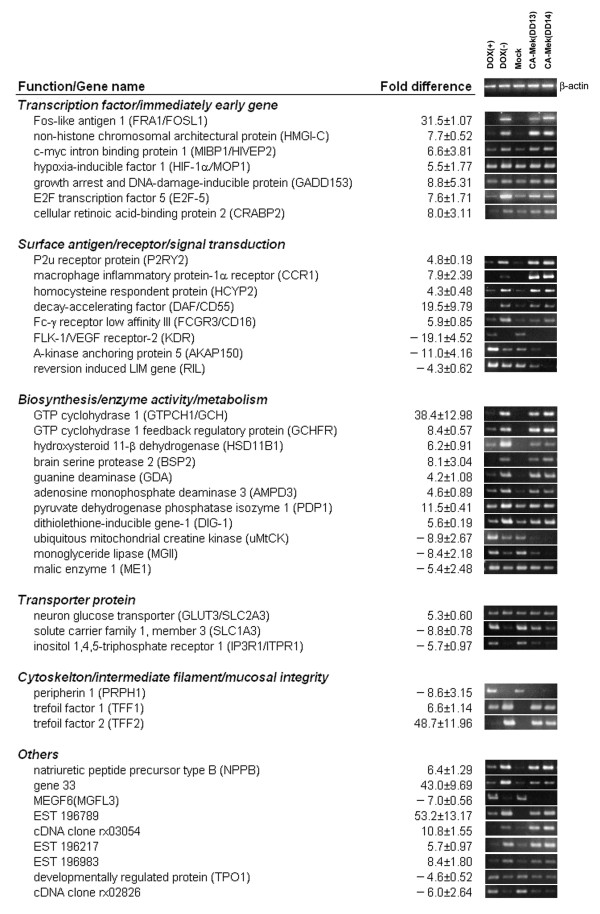
Altered expression levels of caMEK-regulated genes from transcription factor, signal transduction, metabolic, transportation, cytoskeletal, and other pathways. The microarray results from caMEK expressing cells (DOX(-)) compared to normal cells (DOX(+)) are expressed as fold difference ± S.D. Differentially expressed genes were verified through RT-PCR analysis. β-actin was used to indicate equal template in each lane.

To confirm the differential expression of the genes observed from the microarray results, RT-PCR analysis was performed using gene-specific primers and RNA from MEK-inducible RIEtiCAMEK cells in the presence of NaB. Over 97% of all transcripts (113/115) observed by microarray were verified by RT-PCR analysis from the RIEtiCAMEK cells (data not shown). In order to account for the possibility that transcripts were altered by a histone deacetylase (HDAC) inhibitor which could potentially influence global gene expression [30,31], we also determined the gene profile of other caMEK and empty vector transfected cells in the absence of NaB. Therefore, RT-PCR analysis was performed on constitutively expressing caMEK clones (RIEcCAMEK cells; clone DD13, DD14) [8], as well as empty vector transfected cells (RIE-mock cells) in the absence of NaB. We confirmed 69 genes with altered transcription levels in both cell systems induced by caMEK (Figure 1, 2). However, the altered expression of 46 genes was not confirmed in the second cell system. Therefore, these 46 transcripts may not be regulated by caMEK and are possibly influenced by a HDAC inhibitor. The results from both cell systems indicated that 69 genes may be true targets of MEK-activation in RIE cells. The majority of these differentially expressed genes have previously been implicated in cellular transformation or tumorigenesis, including TGF-α and cyclooxygenase-2 (up-regulated genes) as well as DOC-2/DAB2 (down-regulated gene).

### Gene expression analysis in human colon cancers by RT-PCR

**Figure 3:**
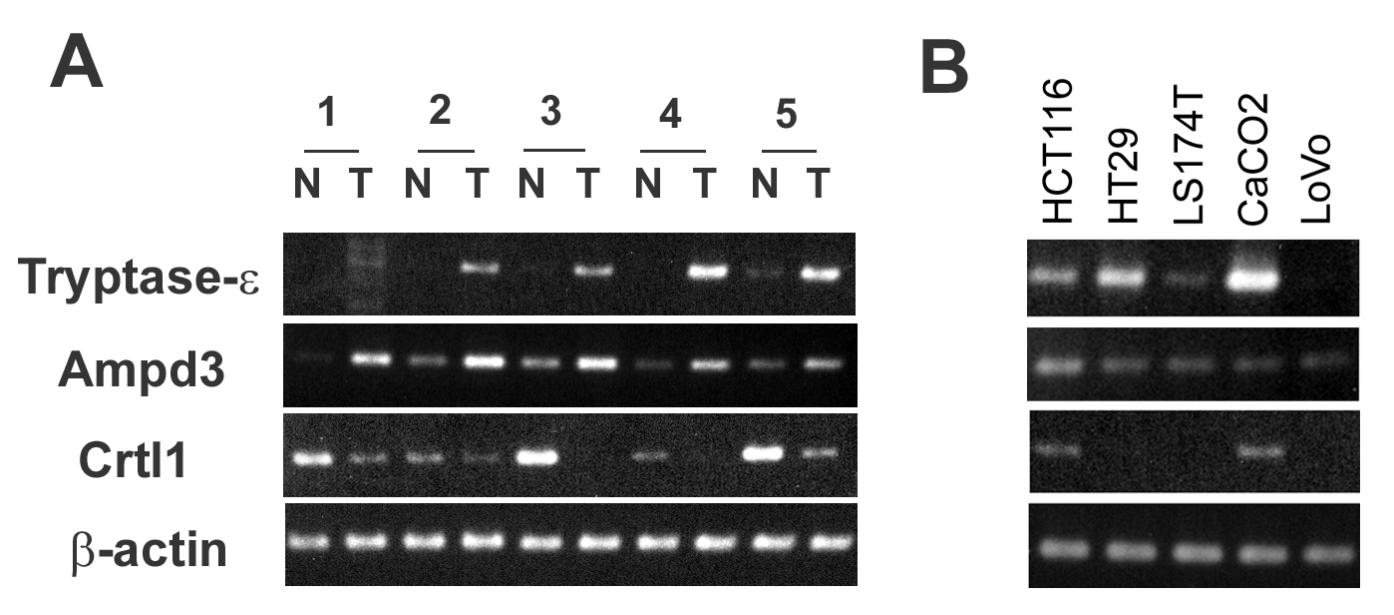
**RT-PCR analysis of human colon cancer tissue and cell lines**. (A) RT-PCR analysis was performed on 5 paired normal and tumor human colon cancer tissues. T indicates tumor tissue and N indicates corresponding normal adjacent mucosa. Gene-specific primers for PCR were designed by MacVector 7 software depending on the information from GeneBank. Amplication of the right target DNA was confirmed by sequence analysis. β-actin was used as an internal control to confirm equal amount of the templates. (B) RT-PCR analysis was performed on 5 human colon cancer cell lines (HCT116, HT29, LS174T, CaCO2, LoVo) with the indicated primer sets.

From the results of above experiments, we searched approximately 69 genes using PubMed (National Center for Biotechnology Information) for their involvement in cellular transformation or human cancer. We found that 8 genes (NPPB, PRSS22, CCR1, CTPCH1, P2RY2, AMPD3, CRTL1, AKAP150) did not have clear involvement. We focused on these 8 genes and performed RT-PCR analysis using the samples from 5 human colon cancer cell lines and human colon cancer tissues (tumor and corresponding adjacent normal mucosa from individual patients). Three novel targets were shown to have altered expression levels (Figure 3A,B). Human tryptase-ε/PRSS22, which is highly homologous to rat brain serine protease bsp2, and adenosine monophosphate deaminase 3 (AMPD3) were up-regulated in all 5 human colon cancer tissues compared to the corresponding normal mucosa. These transcripts were also expressed in several different colon cancer cell lines (4 of 5 and 5 of 5 respectively). Conversely, cartilage link protein 1 (CRTL1) was down-regulated in all 5 human colon cancer tissues and was expressed in only two of 5 colon cancer cell lines.

## Discussion

Recently, we reported that caMEK signaling is highly oncogenic and induces cellular transformation in rat intestinal epithelial cells [8]. We now show,, through the use of microarray analysis, that many genes associated with cellular transformation have altered expression levels following constitutive MEK activation. MEK-ERK signaling is associated with cell migration, invasion, and metastasis [18,19]. Our array results indicate that 10 transcripts associated with cell migration (e.g. MMP10, MMP13, etc) and adhesion (e.g. PTHLH, OPN, etc) have altered expression levels following MEK activation. Additionally, 7 genes known to possess tumor suppressor function (e.g. GPC3/OCI-5, LOT1/PLAGL1/ZAC1, etc) were down-regulated by MEK-activation. Furthermore, several genes that possess anti-apoptotic or chemo-resistant properties were over-expressed in caMEK expressing clones. The altered expression of transcripts was also seen in genes that are involved in growth and proliferation, transcription, signal transduction, biosynthesis, and the cytoskeleton. Together, this data supports our finding that MEK signaling positively regulates transformation in intestinal epithelial cells.

Among the most interesting findings, surface antigen CD44, complement resistance factor CD55/Daf, and secreted phosphoglycoprotein OPN, all of which are known to be implicated in colorectal cancer [25-32], were also up-regulated by caMEK. All of these results suggest the importance of MEK signaling in the intestinal tumorigenesis. Oncogenic transformation of rat intestinal epithelial cells following MEK-activation may depend on the balance between increased transcription of tumor-promoting genes and reduced levels of tumor suppressor genes.

We also have shown three transcripts that may be involved in human colorectal cancer. Of particular interest are the up-regulation of tryptase-ε/PRSS22 and AMPD3, and the down-regulation of CRTL1. Tryptase-ε/PRSS22 is a member of the chromosome 16p13.3 family of human serine proteases that is preferentially expressed by epithelial cells [36]. The tryptase-ε/PRSS22 gene is expressed in the airways in a developmentally regulated manner and is a major product of several different transformed epithelial cell lines [36]. Malignant cells require a range of proteolytic activities to enable growth, survival, and expansion [37]. Tryptase-ε/PRSS22 may play a role in this process. AMPD3 is one of the isoforms of the AMP deaminase family, which converts AMP to IMP and is a diverse and highly regulated enzyme that is a key component of the adenylate catabolic pathway [38]. This enzyme serves to protect the cell against sharp decreases in the adenylate energy charge by removing AMP generated when the rate of utilization of ATP is suddenly increased [39]. In cancer cells, a marked imbalance in the enzymic pattern of purine metabolism is linked with transformation and/or tumor progression [40]. This enzymatic change of purine metabolism seems to be present in transformed intestinal epithelial cells. CRTL1 (also known as a link protein) is a small glycoprotein of the extracellular matrix that was originally identified for its role in stabilizing aggregates of aggrecan and hyaluronan in cartilage [41]. In addition to being expressed in cartilage, CRTL1 is also immunolocalized in several noncartilaginous tissues [41]. A recent study has suggested that CRTL1 may be a down-stream target of β-catenin in intestinal epithelial cells, which has been implicated early in the progression of colorectal epithelial cells to cancer [42]. Therefore, this gene may also serve a role in preventing tumor formation of intestinal cells. This is the first report which indicates the involvement of these three genes in colorectal cancer.

## Conclusion

Although a great body of evidence shows the importance of Ras and its downstream signaling mediators (Raf-MEK-ERK) on colorectal tumor development, the precise role of MEK remains undefined. Our results show that several genes previously known to be implicated in cellular transformation or tumorigenesis were altered following constitutive MEK activation in rat intestinal epithelial cells. Therefore, the MEK-ERK cascade seems to play an important role in intestinal transformation. Also, this is the first report, which indicates the involvement of these three genes in colorectal cancer. Some of the genes acting downstream of this signaling pathway may become useful markers for detection or therapeutic targets for colorectal cancer.

## Methods

### Cell lines and preparation of total RNA

The RIEtiCAMEK cells, RIEcCAMEK cells, and RIE-mock cells have been previously described [8]. Human colon cancer cell lines, HT29, CaCO2, LS174T, HCT116, and LoVo cells were purchased from American Type Culture Collection (ATCC). The cells were maintained in Eagle's minimal essential medium (Invitrogen, Carlsbad, CA) (CaCO2 and LS174T cells), McCoy 5A medium (Invitrogen) (HT29 and HCT116 cells) or Ham's F12 medium (Invitrogen) (LoVo cells) with 10% heat-inactivated fetal bovine serum (FBS) (Hyclone Laboratories, Logan, UT), and 2 mM L-glutamine. Total RNAs were isolated from each cells using TRIzol reagent (Invitrogen), and were purified by the RNeasy mini kit (Qiagen, Valencia, CA) following treatment with DNase I.

### Analysis of gene expression by microarray

Total RNAs were isolated from RIEtiCAMEK cells with/without 2 μg/ml doxycycline (DOX) (BD Bioscience, Palo Alto, CA) following treatment with a histone deacetylase (HDAC) inhibitor (5 mM sodium butyrate (NaB) (Sigma, St.Louis, MO). The HDAC inhibitor can enhance transgene expression under the control of the CMV promoter [26-28], and induces nearly a 3000-fold increase of transgene expression in the cells (data not shown). Samples were sent to Genome Explorations, Inc. (Menphis, TN), where the RNA samples were converted to biotinylated cRNA and hybridized to the Affymetrix (Santa Clara, CA) Rat Genome U34A GeneChip array according to manufacturer's directions. The scanned images were analyzed using Microarray software (Affymetrix). Sample loading and variations in staining were standardized by scaling the average of the fluorescent intensities of all genes on an array to constant target intensity (2500) for all arrays used. The expression data were analyzed as previously described [29]. The signal intensity for each gene was calculated as the average intensity difference, represented by [μ(PM – MM)/(number of probe pairs)], where PM and MM denote perfect-match and mismatch probes. The analysis was performed twice (biological and technical replicates).

### Analysis of gene expression by RT-PCR

Single-stranded cDNA was synthesized using oligo-(dT) primer and Superscript II reverse transcriptase (Invitrogen). PCR reactions were done in 50 μL volumes and amplified for 2 minutes at 94°C for initial denaturation, followed by 20–30 cycles at 94°C for 30 seconds, 50–64°C for 30 seconds, and 72°C for 1 minute (the conditions of reaction cycles and annealing temperatures were optimized for each individual pair of primers). PCR products were separated on 1.6–2.0% agarose gels and visualized by ethidium bromide staining. Amplication of the correct target DNA was confirmed by sequence analysis. Gene-specific primers for PCR products were designed by MacVector 7 software (Accelrys, San Diego, CA) using information from GenBank (NCBI). Gene function annotations were obtained from the Affymetrix web site and/or GenBank. RT-PCR analysis was also performed with samples from human colon cancer cell lines and human colon normal and tumor matched cDNA pair panels (BD Bioscience). β-actin was used as an internal control to confirm equal amounts of template.

## Abbreviations

caMEK, constitutively activated MEK; COX-2, cyclooxygenase-2; DOX, doxycycline; ERK, extracellular signal-regulated protein kinase; HDAC, histone deacetylase; MAPK, mitogen-activated protein kinase; MEK, mitogen-activated protein kinase kinase; NaB, sodium butyrate

## Authors' contributions

KK carried out the molecular genetic studies, designed the study, and drafted the manuscript. MJ analyzed the microarray results. MO and YH carried out the RT-PCR analysis. FGB, MO, SW, and RND conceived the study, participated in its design and coordination and helped to draft the manuscript. All authors read and approved the final manuscript.

## Grant support

The United State Public Health Services Grants DK 47297, P30CA-68485, DK 62112, and PO1CA-77839 (RND), and Research Fellowships of Uehara Memorial Foundation (KK)
